# Marfanoid phenotype with intellectual disability associated with *NKAP* mutation: a case report

**DOI:** 10.1186/s13256-026-05984-2

**Published:** 2026-03-31

**Authors:** Alla N. Semyachkina, Ekaterina A. Nikolaeva, Daria Iu. Gritsevskaya, Victoria Yu. Voinova, Rabiat G. Kuramagomedova, Sergei V. Bochenkov, Olga S. Groznova, Mariia A. Parfenenko, Olga V. Kalashnikova, Evgenia A. Isupova

**Affiliations:** 1 Veltischev Research and Clinical Institute for Pediatrics and Pediatric Surgery (Veltischev Institute) of the Pirogov Russian National Research Medical University, Moscow, Russia; 2 Institute of Continuing Education and Professional Development, Pirogov Russian National Research Medical University, Moscow, Russia; 3 Charity Fund for Medical and Social Genetic Aid Projects “Life Genome”, Moscow, Russia; 4 Petersburg State Pediatric Medical University, St. Petersburg, Russia

**Keywords:** Marfanoid phenotype, Hackman–Di Donato syndrome, *NKAP*

## Abstract

**Background:**

Hackman–Di Donato syndrome (Online Mendelian Inheritance in Man no. 301,039), caused by mutations of the *NKAP* gene, located in Xq24, is a rare genetic disorder characterized by a Marfanoid phenotype, intellectual disability, and abnormalities of the musculoskeletal system. This condition has only been described in ten patients and two fetuses, who were identified owing to severe heart defects. All previously reported patients had mutations located in exons 8 and 9 of *NKAP*.

**Case presentation:**

The proband, a 12-year-old Slavic male individual, presented with a Marfanoid habitus, tall stature, dolichostenomelia, scoliosis of the lumbar spine, moderate pectoral deformity, plano-valgus position of the feet, joint hypermobility, habitual dislocation of the right shoulder joint, arachnodactyly of the hands and feet, elongated face, muscle hypotonia, absence epilepsy, and borderline intelligence. Whole-genome sequencing revealed a missense variant NM_024528.4 chrX:119936358-T > G ENST00000371410.5:c.612A > C ENST00000371410.5 (ENSP00000360464.3):p.Arg204Ser in exon 4 of *NKAP*, classified as a variant of unknown significance. On the basis of clinical and genomic data, the child was diagnosed with X-linked syndromic intellectual developmental disorder, Hackman–Di Donato type. The pathogenicity of the nucleotide variant of exon 4 of *NKAP* requires further analysis.

**Conclusion:**

The observed case may indicate greater genetic heterogeneity of Hackman-Di Donato syndrome, linking it to mutations not only in exons 8 and 9 but also in other exons of *NKAP*, though more data on the variant effect, as well as the significance of exon 4 of *NKAP* functioning is needed. Additionally, the case illustrates the need to test male patients with a Marfanoid phenotype and intellectual disability for mutations in *NKAP*.

## Introduction

In the past few decades, a series of phenotypes were described that clinically resemble Marfan syndrome but are associated with mutations of genes other than *FBN1*. Among them are Loeys–Dietz syndrome, Stickler syndrome, Lujan–Fryns syndrome, and other conditions. The growing use of DNA sequencing methods in clinical practice has enabled the identification of many more similar disorders. Thus, in 2019, a new Marfan-like syndrome, additionally presenting with intellectual disability and mutations of the *NKAP* gene, was reported by Fiordaliso *et al*. [1].

*NKAP* is located in Xq24. The gene product is the nucleoplasmic protein NKAP (NFKB-activating protein), which is a highly conserved protein involved in transcriptional repression, T cell development, maturation and survival of adult hematopoietic stem cells, and RNA splicing and processing [2, 3]. NKAP contains 415 amino acids and has a molecular weight of 47 kDa. The protein is expressed ubiquitously, with the greatest rates of expression observed in the spleen, skin, testes, kidneys, and lungs. In the cell nucleus, NKAP is localized in clusters of interchromatin granules and functions as a speckle protein—a component of the spliceosome. The structure of NKAP includes an N-terminal RS domain (motif), a main (basic) domain, and a C-terminal DUF926 domain. NKAP is known to interact with RNA-binding proteins, RNA helicases, and splicing factors [3].

In 2019, Fiordaliso *et al*. published the results of a study of ten male patients from eight families in whom missense mutations were found in *NKAP* [1]. The disorder was designated as intellectual developmental disorder, X-linked syndromic, Hackman–Di Donato type (Hackman–Di Donato syndrome [HDDS])—owing to the fact that one out of ten patients was identified within the cohort of patients clinically diagnosed with Lujan–Fryns syndrome—described by Hackmann *et al*. [4]. The age of the patients ranged from 4 to 21 years; they were of different ethnic backgrounds. All of them had intellectual disability, abnormalities of the musculoskeletal system, and a Marfanoid phenotype. Among other clinical features, the following were mentioned: aggressive behavior, features of the facial phenotype (long narrow face, protruding ears, epicanthus, high palate, among others), and abnormalities of the cardiovascular system and genitalia.

Pedigree analysis showed that three patients (one pair of siblings and an unrelated patient) inherited the disease from asymptomatic carrier mothers. In one pedigree, two siblings appeared to have inherited the mutation due to maternal germline mosaicism. In other cases, the disease was caused by de novo mutations in *NKAP*.

In ten identified patients, five different missense mutations were identified, of which the c. 998G > A (p.Arg333Gln) mutation was found in six cases. All pathogenic nucleotide variants were located in exons 8 and 9, encoding the C-terminal part of NKAP.

In 2024, Xu *et al*. [5] reported two cases of prenatal diagnosis of severe congenital heart defects (ventricular septal defect, pulmonary stenosis, aortic dextraposition) in male fetuses (siblings) with the c.988C > T (p.Arg330Cys) mutation in *NKAP*, previously described by Fiordaliso *et al*. [1] The mother and other female maternal relatives were found to be carriers of the mutation. The variant was absent in male maternal relatives. Both pregnancies were terminated. In addition to heart defects, brain abnormalities were detected in the fetuses: enlargement of the posterior horns of the lateral ventricles (in one) and cysts of the choroid plexus of the ventricles of the brain (in one). The authors confirmed the pathogenic significance of the c.988C > T variant and concluded that the clinical characteristics of the presented cases were consistent with HDDS.

Thus, there are currently 12 reported cases of HDDS. We present our own clinical observation of a child diagnosed with the same disorder on the basis of clinical and genomic data.

## Case report

We report the case of a 12-year-old Slavic male individual who was born to young (25 and 27 years old), healthy parents in their first pregnancy. The family includes a younger male sibling aged 8 years, with similar clinical features. At the 32nd week of gestation, the fetus was diagnosed as breech and at the 40th week of pregnancy, a planned caesarean section was performed. The newborn’s Apgar score was 8/9 points. Body weight at birth was 3230 g, and body length was 55 cm. Early psycho-motor and speech development corresponded to age: He began to support his head at 2 months, sit at 7 months, and walk at 14 months. He began to use distinct words at the age of 1 year, and phrase speech developed at the age of 2 years.

At the age of 3 years, after suffering lacunar tonsillitis, the proband began experiencing pain in the knees and swelling of the ankle joints. On the basis of those symptoms, the patient was diagnosed with juvenile arthritis and prescribed nonsteroidal anti-inflammatory drugs, which the proband took for 7 years. At the age of 10 years, the diagnosis of juvenile arthritis was withdrawn—the inflammatory nature of the disease was ruled out and further use of nonsteroidal anti-inflammatory drugs was deemed unnecessary and was discontinued.

At the age of 5 years, a few days after a planned revaccination of diphtheria–tetanus–whole-cell pertussis vaccine and an acute respiratory viral infection with febrile temperature, the proband first developed short (5–13 seconds) “freezes.” The frequency of attacks reached 50–60 per hour. The patient was diagnosed with absence epilepsy and prescribed anticonvulsants (initially valproic acid [maximum 500 mg/day], with ethosuximide [max. 500 mg/day] being additionally prescribed 3 months later). A year later, valproic acid was discontinued. Remission has been maintained since the age of 11 years, and the patient is currently receiving 1000 mg/day of ethosuximide.

Meanwhile, the patient underwent genetic and biochemical testing. Cytogenetic analysis revealed a normal male karyotype (46,XY), while blood and urine tests for amino acids, acylcarnitines, and organic acids allowed us to rule out hereditary aminoacidopathy, organic aciduria, and mitochondrial beta-oxidation disorders.

**Fig. 1:**
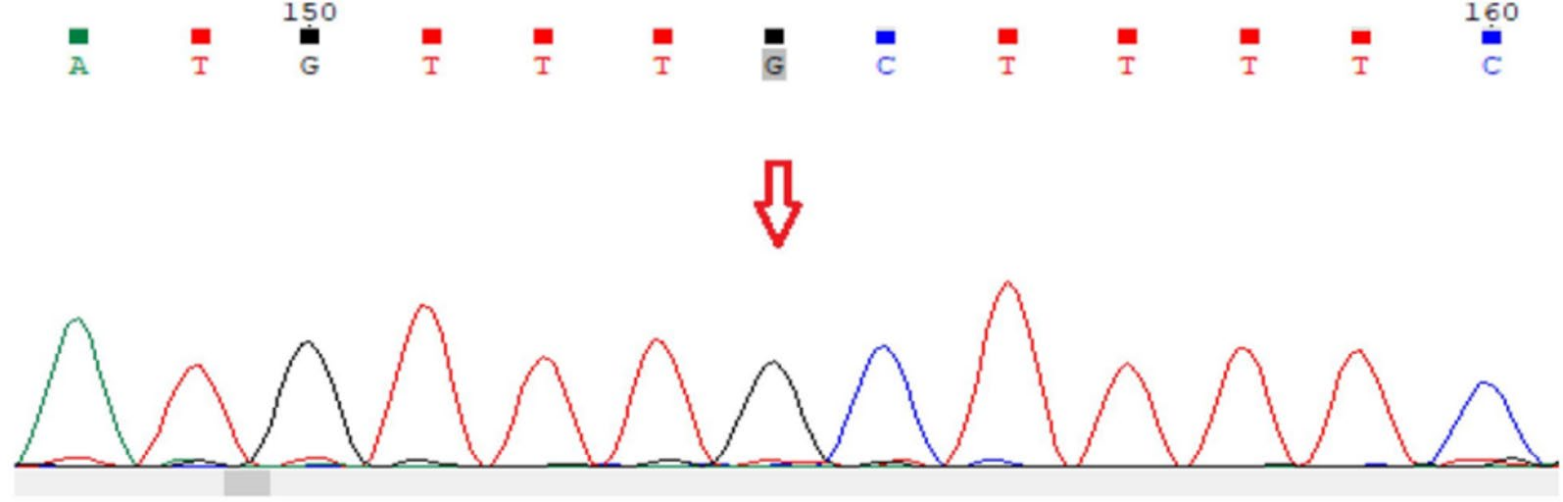
The nucleotide variant NM_024528.4 chrX:119936358-T > G ENST00000371410.5:c.612A > C ENST00000371410.5 (ENSP00000360464.3):p.Arg204Ser in exon 4 of the *NKAP* gene found in hemizygous state. The nucleotide change is indicated by an arrow

**Fig. 2:**
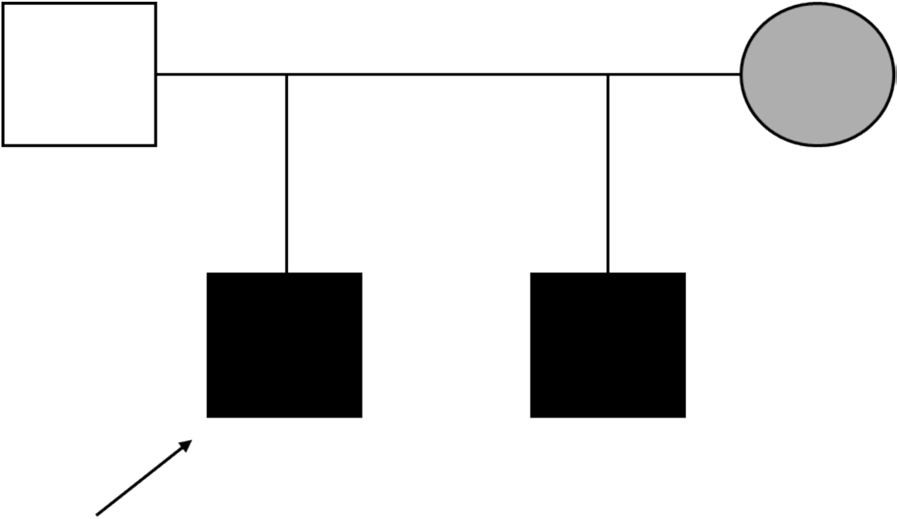
The pedigree. The proband is indicated with an arrow. The black square signifies affected, the gray circle signifies unaffected heterozygous carrier, and the white square signifies healthy noncarrier

The proband then underwent whole-genome sequencing (WGS), which showed a previously undescribed missense variant NM_024528.4 chrX:119936358-T > G ENST00000371410.5:c.612A > C in exon 4 of *NKAP* (Fig. 1), leading to the substitution of the amino acid arginine with serine at position 204 (ENST00000371410.5 (ENSP00000360464.3):p.Arg204Ser). Hemizygous variants in *NKAP* have been described in male patients with HDDS. The variant is not registered in the gnomAD 3.1.2 test sample and can be classified as a variant of unknown clinical significance on the basis of the following criteria: PP1: co-segregation with disease in multiple affected family members; PM2: absent or very low frequency in population databases; PP4: the patient’s phenotype is highly specific for a single genetic etiology. Using Sanger sequencing, the presence of the identified variant was confirmed in the proband and his affected male sibling in a hemizygous state and discovered in the mother in a heterozygous state. The pedigree is shown in Fig. 2. No other clinically significant gene variants were found.

The 12-year-old male proband was admitted to the Department of Clinical Genetics of the Veltishchev Institute. His parents reported the following complaints regarding his condition: delayed speech development, seizures (in remission for 1 year), memory impairment, decreased visual acuity, pain in the knee and ankle joints, habitual dislocation of the right shoulder joint.

**Fig. 3:**
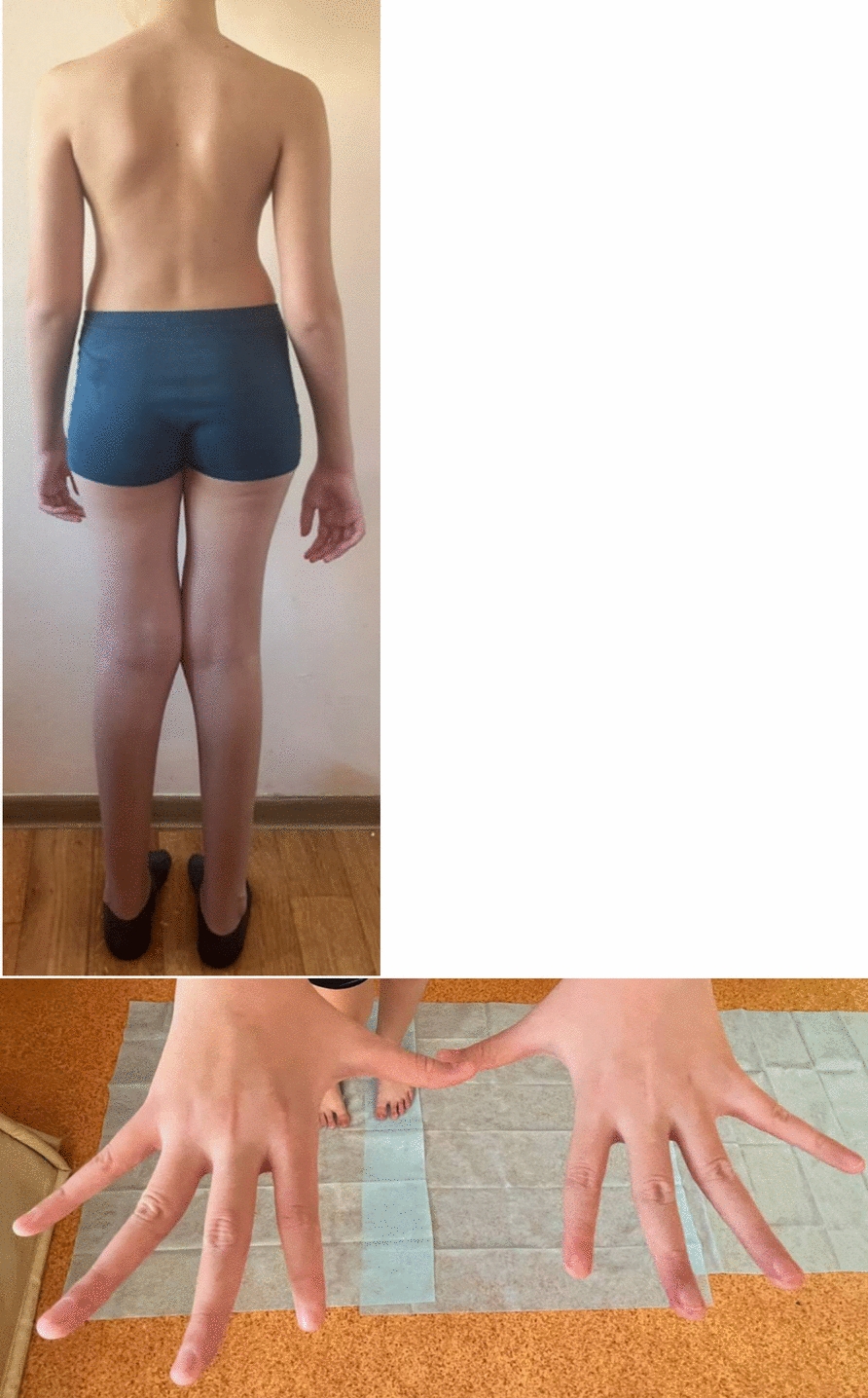
The proband, 12 years old, with Hackman–Di Donato syndrome: Marfanoid phenotype, long extremities, scoliosis (**a**); arachnodactyly (**b**)

Upon examination, indicators of physical development were disharmonious: body length was 167.5 cm (above 97th percentile), body weight was 48 kg (75–90th percentile), and head circumference measured 53.5 cm, which fell within the average values (25–50th percentiles). The proband’s phenotype included: high stature, dolichostenomelia, left-sided scoliosis of the lumbar spine of 1–2°, moderate pectoral deformity, plano-valgus position of the feet, joint hypermobility, habitual dislocation of the right shoulder joint, arachnodactyly of the hands and feet, and an elongated face. The following minor anomalies of the phenotype were observed—epicanthus, high palate, retrognathia, short philtrum, and protruding auricles (Fig. 3). According to physical data, no changes in internal organs were detected. The genitalia were formed normally, typical for a biological male. The proband attends a regular school but requires significant parental assistance throughout his education. Clinical and biochemical blood and urine tests showed normal values. The electrocardiogram showed moderate sinus arrhythmia, with periods of moderate tachycardia, as well as increased electrical activity of the left ventricle. According to the echocardiogram, mitral valve prolapse (3 mm) with mild mitral regurgitation was observed. Blood pressure monitoring for 24 hours showed normal values.

Diffuse epileptiform activity was recorded during video-electroencephalogram monitoring during sleep. Ultrasound of the abdominal organs and kidneys revealed no abnormalities. X-rays of the hands showed a delay in bone age of 2–2.5 years; densitometry did not detect a decrease in total bone mineral density according to the *Z*-score.

Apart from the state of remission of childhood absence epilepsy, the following neurological symptoms were noted: gait disturbances, difficulties when walking up stairs, and the use of auxiliary techniques when getting up from the floor. The intellectual quotient (IQ) test score of the proband was 75, which corresponded to the borderline level of intellectual development (70–79 range) according to the Wechsler scale. Vision testing showed mild myopia. The full timeline for the patients’ symptoms (diagnoses) and interventions is presented in Table 1.

**Table 1:** The timeline of key diagnostic and clinical events

	Early childhood (0–3 years old)	Preschool age (3–6 years old)	Prepuberty (6–12 years old)	At the time of examination
Symptoms	Arthropathy following tonsillitis at 3 years of age; initially diagnosed with juvenile arthritis	Absence epilepsy begat at the age of 5 years, following vaccination and viral infection	Seizure remission achieved Diagnosis of juvenile arthritis disproven	HDDS formally diagnosed on the basis of clinical and genetic evidence
Interventions	Prescribed nonsteroidal anti-inflammatory drugs	Prescribed valproic acid (maximum 500 mg/day), and ethosuximide (maximum 500 mg/day) at 5 years of age, valproic acid discontinued at 6 years of age	Nonsteroidal anti-inflammatory drugs discontinued WGS showed c.612A > C in exon 4 of NKAP	Sanger sequencing determined the presence of c.612A > C in affected brother (hemizygous) and unaffected mother (heterozygous)

Thus, the following clinical features were identified in the proband: tall stature, dolichostenomelia, arachnodactyly, left-sided scoliosis of the lumbar spine of 1–2°, flat-valgus position of the feet, joint hypermobility, habitual dislocation of the right shoulder joint, elongated face, mild myopia in both eyes (OU), borderline level of intellectual development, and absence epilepsy (in remission). In the process of differential diagnosis, several conditions characterized by a Marfanoid phenotype and hypermobility spectrum disorders were considered. On the basis of clinical data, as well as the results of WGS, the proband was diagnosed with HDDS. Two symptoms—childhood absence epilepsy (in remission) and a history of arthropathy of unknown origin—have not been previously associated with HDDS.

Genetic counseling was provided to the family, discussing the X-linked inheritance pattern, recurrence risks (50% for subsequent male children), and the availability of carrier testing for female relatives (none at present).

The following recommendations for clinical surveillance were given to the patient (patient’s family):

- Regular cardiology follow-up with annual echocardiograms to monitor the mitral valve prolapse and aortic root diameter.
- Orthopedic care for scoliosis, joint hypermobility, and habitual dislocation, including physical therapy.
- Continued neurology management with routine electroencephalography (EEG) monitoring to manage his absence epilepsy.
- Ophthalmological examinations to monitor his myopia.
- Neuropsychological assessment and ongoing educational support owing to his borderline intellectual functioning.

## Discussion

HDDS, caused by mutations in *NKAP* and inherited in an X-linked recessive manner, is a very rare hereditary disease. To date, only 12 patients have been described, including 2 cases of prenatal diagnosis in fetuses [1, 5]. It appears that our proband is the 13th.

In the previously reported patients, five missense mutations of *NKAP* were found, located in exons 8 and 9, which encode the C-terminal domain of the NKAP protein. The pathogenic effect of mutations on the transcriptome has been proven. The experiment established that mutations affecting this region led to the formation of a truncated protein and severe developmental defects. Of the five, four mutations led to the substitution of arginine with glycine (seven cases from five families), cystine (three cases from two families) or histidine (one case); one mutation resulted in the substitution of isoleucine with threonine [1, 5]. In our patient, the identified mutation NM_024528.4 chrX:119936358-T > G ENST00000371410.5: c.612A > C ENST00000371410.5 (ENSP00000360464.3): p.Arg204Ser led to the substitution of arginine by serine at position 204 of NKAP. The mutation is located in exon 4 of *NKAP* and is absent in the gnomAD 3.1.2 control sample, but is classified as a variant of unknown clinical significance. At this time, there is no laboratory evidence of the pathogenicity of this nucleotide variant, and there is no reliable information about the functional significance of exon 4 of *NKAP*.

Studies have established that NKAP is a regulator of gene expression, but it does not have a specific DNA-binding domain and likely acts as part of larger molecular complexes. The C-terminal domain of NKAP binds to histone deacetylase 3 (HDAC3), which is involved in modifying gene expression [2, 6, 7]. NKAP also interacts with CIR (CBF1-interacting corepressor), which is part of the corepressor complex of the Notch signaling pathway; the latter serves as a regulator of cellular homeostasis, cell growth, differentiation, among others. Apparently, NKAP, through interaction with CIR, modulates Notch-mediated transcription. It has been established that CIR binds not to the C-terminal domain, which is encoded by exons 8–9 of *NKAP*, but to another domain, which appears to be encoded by exons 3 or 4 of the gene [6].

Common clinical manifestations in individuals with *NKAP* mutations reported in the literature include the following: developmental delay, behavioral disturbances, muscle hypotonia, joint hypermobility, Marfanoid habitus with tall stature and arachnodactyly, and features of the following facial phenotype: epicanthus, long narrow face with hypoplasia of the midsection, short philtrum, and dysplastic ears. Comparison with our patient demonstrates great similarity in the main clinical symptoms (Table 2). The differences included the proband’s lack of aggressive behavior, camptodactyly, and abnormalities of the genitalia. However, as presented in the table, these signs are characteristic of no more than half of the patients. Additionally, our patient presented with absence epilepsy and arthropathy of unknown origin with a history of swelling and pain.

**Table 2:** Comparison of the clinical features observed in ten patients with Hackman-Di Donato syndrome previously reported by Fiordaliso *et al*. and our proband

Clinical features	Previously reported with Hackman-Di Donato syndrome, *n* = 10	The proband
Disorders of the central nervous system:	10	+
Developmental delay/intellectual disability	10	+
Aggressive behavior	5	−
Epilepsy	0	+
Disorders of the musculoskeletal system:	10	+
Tall stature	10	+
Scoliosis	6	+
Pectoral deformity (pectus excavatum/pectus carinatum)	5	+
Long extremities	9	+
Joint hypermobility	9	+
Habitual dislocation of the shoulder joints	0	+
Pain and swelling of the joints	0	+
Camptodactyly	4	−
Arachnodactyly	8	+
Muscle hypotonia	10	+
Disorders of the cardiovascular system:	7	+
Atrial/ventricular septal defect	2	−
Patent ductus arteriosus	1	−
Dilatation of the aorta	1	−
Mitral valve prolapse with associated regurgitation	3	+
Abnormalities of the genitalia:	5	−
Cryptorchidism	2	−
Hypospadias	2	−
Micropenis	1	−
Minor anomalies of the phenotype:	10	+
Short philtrum	7	+
Hypoplasia of the bottom half of the face	7	+
Large, protruding auricles	9	+
Epicanthus	9	+
“Gothic” palate	10	+
Narrow, elongated face	8	+

The number in the second column refers to the number of patients who had the listed clinical feature, while the ± in the third column shows whether the clinical feature was present in our proband

The phenotypic similarity between patients with a mutation in *NKAP* and patients with other conditions characterized by a Marfanoid phenotype is noteworthy. The similarity with Marfan syndrome can be seen, primarily in the same type of skeletal abnormalities—tall stature, asthenic physique, scoliosis, pectoral deformity, long thin extremities, arachnodactyly, and narrow facial skeleton [8]. However, HDDS differs from Marfan syndrome mainly owing to the involvement of the central nervous system, the absence of lens subluxation, and severe damage to the aorta (only moderate dilatation of the aortic root was detected in one out of ten patients). The joint hypermobility characteristic of patients with HDDS necessitates differentiation with Ehlers–Danlos syndrome, and in our proband, the similarity was strengthened by the presence of habitual dislocation of the shoulder joint. However, previously, other patients, as well as our proband did not show increased skin extensibility and fragility of blood vessels and tissues characteristic of Ehlers–Danlos syndrome. The need to differentiate this condition from Lujan–Fryns syndrome is evidenced by the fact that the first patient with HDDS was genetically identified in a group of patients clinically diagnosed with that disorder. Lujan–Fryns syndrome may be differentiated by the following features—macrocephaly, a nasal voice, and a violation of the structure of the teeth (dental crowding, double row of teeth).

The clinical manifestations of HDDS that are characteristic of connective tissue disorders likely arise from the common elements in their pathogenesis. According to Fiordaliso *et al*., *NKAP*plays a critical role in transcriptional regulation during neuronal and connective tissue development [1]. The Notch signaling pathway has been shown to be a transcriptional target of NKAP. It has been suggested that *NKAP* mutations indirectly, through Notch, affect the TGF-β signaling pathway, which is involved in the regulation of extracellular matrix gene expression [6, 9]

## Conclusion

Thus, the clinical features of our patient correspond to those observed in previously reported patients with HDDS. However, the pathogenic significance of the nucleotide variant chrX:119936358-T > G ENST00000371410.5: c.612A > C ENST00000371410.5 (ENSP00000360464.3): p.Arg204Ser in exon 4 of *NKAP* identified in the patient currently requires further analysis. Our observation may indicate greater genetic heterogeneity of HDDS; however, with the variant affect, as well as the function of exon 4 of *NKAP* being unknown, more data are required to confidently link HDDS to mutations not only in exons 8–9 but also in other exons of *NKAP*.

HDDS is not yet familiar to pediatricians and clinical geneticists. Male patients with a Marfanoid phenotype, especially those with cognitive impairment, should be tested for mutations in *NKAP*.

## Data Availability

All relevant data are included in the manuscript.
